# Health Tract, No. 243

**Published:** 1866-06

**Authors:** 


From the Boston Watchman and Reflector, by Dr. W. W. Hall, M.D.

HEALTH TRACT, NO. 243.

### EXTEMPORANEOUS SURGERY.

Even young chidren should be taught how to act in some of the accidents of life which require surgical skill. The arteries of the body carry the life's blood from the heart. If one of these is ruptured from any cause, and the blood is allowed to escape, the man will die within a few minutes sometimes, when with the aid of a stick and a string or handkerchief, either of which are almost always at hand, his life might be saved. If the severed artery is in the leg or arm, and there is no string at hand, tear a strip from any part of the clothing, tie it loose around the limb, pass the stick between the skin and the string and twist it round until the bleeding ceases. If a vein is wounded or cut, apply the dust from a tea canister or common cobweb ; or even without these, wrap a strip of cotton cloth around moderately tight, and then another piece around that ; if the bleeding does not cease, let cold water run on the wound until it does, or until a physician arrives. But it is of vital importance to remember that the artery sends out blood by spurts or jets, and of a bright red character. If the blood comes from a vein, it flows slowly and evenly, and is of a dark red. But these directions will do no good unless it is specially noted that if the blood comes from an artery, the application of the string must be made above the wound, that is, between the wound and the heart ; if a vein has been wounded, and the same appliances are needed they must be made below the wound, or between the wound and the extremities.

If an artery is cut in a part of the body where a string cannot be applied, hard pressure with the thumb at a spot about where the string would have been applied may save life.

If stung or bitten by insect, snake or animal, apply spirits of hartshorn very freely with a soft rag, because it is one of the strongest of alkalies, and is familiar to most persons. The substance which causes the so-called poison from bites or stings, is, as far as is ascertained, generally acid. Hence the hartshorn antagonizes it in proportion to the promptitude with which it is applied. If no hartshorn is at hand, pour a cup of hot water on a cup of cooking soda or saleratus, or even the ashes of wood just from the stove or fireplace, because all these are strong alkalies, and hartshorn is only best because it is the strongest. There is no conclusive evidence to believe that burning or cutting out a bite has ever done the slightest good. The proof adduced to show that they have been effectual is wholly of a negative character, and, therefore, not decisive.

